# Risk of dementia in primary aldosteronism compared with essential hypertension: a nationwide cohort study

**DOI:** 10.1186/s13195-023-01274-x

**Published:** 2023-08-11

**Authors:** Namki Hong, Kyoung Jin Kim, Min Heui Yu, Seong Ho Jeong, Seunghyun Lee, Jung Soo Lim, Yumie Rhee

**Affiliations:** 1 Department of Internal Medicine, Severance Hospital, Endocrine Research Institute, Yonsei University College of Medicine, 50-1 Yonsei-Ro, Seodaemun-Gu, Seoul, 03722 Republic of Korea; 2 Department of Internal Medicine, Korea University College of Medicine, Seoul, Republic of Korea; 3 SENTINEL Team, Division of Endocrinology, Department of Internal Medicine, Yonsei University College of Medicine, Seoul, Republic of Korea; 4 Department of Neurology, Inje University Sanggye Paik Hospital, Seoul, Republic of Korea; 5 Department of Internal Medicine, Wonju Severance Christian Hospital, Yonsei University Wonju College of Medicine, 20, Ilsan-Ro, Wonju-Si, Gangwon-Do 26426 Republic of Korea

**Keywords:** Hyperaldosteronism, Hypertension, Dementia, Alzheimer, s disease, Vascular dementia, Adrenalectomy, Mineralocorticoid receptor antagonists

## Abstract

**Background:**

Although hypertension is a critical risk factor for dementia, the association between primary aldosteronism (PA) and dementia has been scarcely reported. We aimed to investigate whether the risk of dementia in patients with PA was elevated compared with patients with essential hypertension (EH).

**Methods:**

From the National Health Insurance Claim database in Korea (2003–2017), 3,687 patients with PA (adrenalectomy [ADX], *n* = 1,339, mineralocorticoid receptor antagonist [MRA] *n* = 2,348) with no prior dementia were age- and sex-matched at a 1:4 ratio to patients with EH (*n* = 14,741). The primary outcomes were all-cause dementia events, including Alzheimer’s disease, vascular dementia, or other dementia combined with a prescription of one or more medications for dementia (donepezil, galantamine, memantine, or rivastigmine). Multivariable Cox regression models were used to evaluate the hazard ratios (HRs) and 95% confidence intervals for the outcome incidence rates between patients with PA and their EH matches.

**Results:**

During a median follow-up of 5.2 years, there were 156 cases of all-cause dementia (4.2%), 140 cases of Alzheimer's disease (3.8%), and 65 cases of vascular dementia (1.8%). Compared with EH, the risk of all-cause dementia was increased in treated PA (unadjusted hazard ratio [HR] 1.26; *p* < 0.011). Among PA, MRA group had higher risks of all-cause dementia, especially vascular dementia, adjusted for age, sex, income, comorbidities, and concurrent medication (adjusted HR 1.31; *p* = 0.027 and adjusted HR 1.62; *p* = 0.020, respectively) compared to EH. ADX group seemed to have a lower dementia risk than the EH group, but there was no statistical significance after full adjustment. This trend became more prominent when the dementia risks were evaluated from the time of hypertension diagnosis rather than treatment initiation for PA.

**Conclusion:**

The findings of this cohort study suggest that PA, especially the MRA group, is associated with an increased risk of dementia. Monitoring cognitive function in PA patients even after treatment initiation might be warranted to prevent dementia.

**Supplementary Information:**

The online version contains supplementary material available at 10.1186/s13195-023-01274-x.

## Background

Primary aldosteronism (PA) is the most common cause of secondary hypertension that is characterized by excessive autonomous aldosterone production [1]. The prevalence of PA is currently reported as 6 to 8% among consecutive patients with hypertension in various care settings, with a prevalence as high as 20% in resistant hypertension [2]. PA is associated with a higher risk of long-term complications than essential hypertension (EH). These include atherosclerotic cardiovascular events, atrial fibrillation (AF), heart failure, a higher prevalence of diabetes mellitus (DM), and chronic kidney disease compared to EH, which poses substantial health and economic burdens [3–5].

Hypertension is a well-established modifiable risk factor for dementia, a progressive disease characterized by a decline in memory and cognitive function [6]. Several community-based cohort studies further suggested that persistent midlife hypertension is associated with an increased risk of dementia in later life [7]. Potentially modifiable risk factors, including less education, low social contact, hearing impairment, smoking, excessive alcohol consumption, and DM, have been reported. Furthermore, recent clinical guidelines for dementia endorse the use of anti-hypertensive treatment as the only known effective preventive medication for dementia [8]. Considering the difficulties in blood pressure control despite the use of multiple anti-hypertensive medications and co-existing conditions such as DM in patients with PA, the risk of dementia may be higher in PA than in EH. Accordingly, a more tailored strategy to prevent later life dementia in patients with hypertension due to PA will be needed.

To date, no longitudinal or cross-sectional studies have been conducted that directly investigate the connection between primary aldosteronism and the risk of dementia. However, a case report on what is referred to as Conn's dementia, coupled with the established hypertensive effects of primary aldosteronism, provides a possible association with Binswanger's dementia—a subtype characterized by white matter lesions [9]. Furthermore, a recent study has elucidated the relationship between elevated plasma aldosterone levels and white matter lesions in patients with hypertension. This study provides supportive evidence regarding aldosterone's role in cerebral changes that are relevant to dementia [10]. In addition, animal studies have highlighted the association between the activation of the Renin–Angiotensin–Aldosterone System (RAAS) and cognitive impairment [11]. Notably, RAAS operates not only systemically but also locally within organs, including the brain. This localized activity of RAAS is critical in understanding its role in organ damage. These insights underscore the significance of further investigating the implications of primary aldosteronism on dementia risk.

This study therefore aimed to investigate the risk of dementia in individuals with PA, grouped by adrenalectomy (ADX) or mineralocorticoid receptor antagonist (MRA) treatment, compared to age- and sex-matched EH controls using a nationwide cohort. We also sought to explore potentially vulnerable subgroups for the onset of dementia in patients with PA.

## Methods

### Data source

This study used the Korean National Health Insurance Service (NHIS) database, consisting of 51.5 million Korean inhabitants from 2002 to 2017. South Korea has a nationwide health system covering approximately 97% of the population to facilitate reimbursements, with all medical institutions submitting healthcare utilization-related information. The database contains medical and pharmaceutical claim records, including disease diagnosis codes (International Classification of Disease, 10th revision [ICD-10]), medical procedure and hospital admission information, prescribed drugs, health examination data such as anthropometric measures, and death records. This detailed data resource was described in previous studies [12].

This study was approved by the Institutional Review Board of Wonju Severance Christian Hospital (IRB number: CR318340). Informed consent was waived because data from the NHIS cohort were fully anonymized and de-identified for the analysis.

### Study subjects

**Fig. 1:**
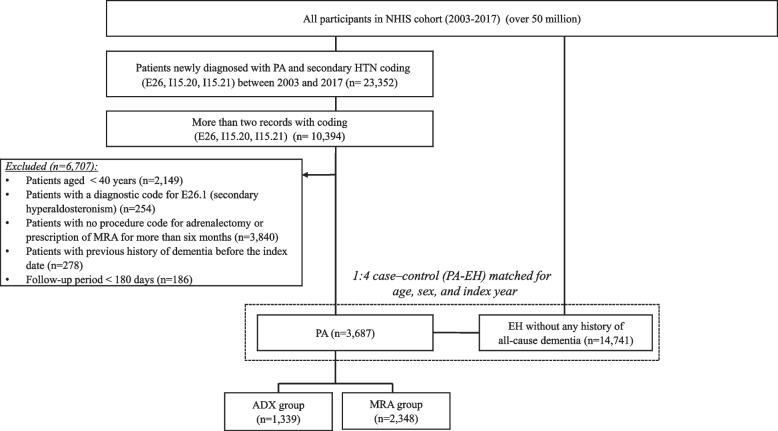
Flow diagram of participant selection. Abbreviations: ADX indicates adrenalectomy; EH, essential hypertension; HTN, hypertension; MRA, mineralocorticoid receptor antagonist; PA, primary aldosteronism

The process of selecting study participants is demonstrated in Fig. 1. To identify patients with likely PA, we used operational definitions based on a combination of diagnostic, procedure, and medication codes. These operational definitions were validated using a multicenter cohort of 920 patients in our previous research [13]. The validation cohort consisted of patients diagnosed with at least one diagnosis code for PA, where 385 (41.9%) of these patients had a biochemically confirmed diagnosis of PA, ascertained through an electronic medical record review. From the original NHIS database, patients diagnosed as having PA with ICD-10 diagnostic codes indicating a primary or secondary diagnosis of E26 (hyperaldosteronism), I15.20 (hyperaldosteronism from adrenal adenoma), or I15.21 (hyperaldosteronism from bilateral adrenal hyperplasia) more than twice between January of 2003 and December of 2017 were selected. Among them, patients who had received adrenalectomy (unilateral or bilateral) or a prescription of MRA for ≥ 180 days were considered as part of the PA group. This approach allowed us to maintain a high level of sensitivity (93.5%) and area under the receiver operating characteristics curve (0.84). The positive predictive value was found to be 73.0% in our previous study [13].

Patients who were younger than 40, had an ICD-10 diagnostic code of E26.1 (secondary hyperaldosteronism) at least once, or had a follow-up period of less than 180 days were excluded. In addition, to detect newly developed dementia events, patients with any previous diagnoses of dementia before study entry were excluded. Subsequently, among PA group patients, those who underwent ADX were assigned to the ADX group, while those who had been prescribed MRA for ≥ 180 days without undergoing any ADX were assigned to the MRA group. Meanwhile, patients with ICD-10 or any diagnostic code for I10 at least twice a year with antihypertensive drug prescriptions for > 180 days (see Supplementary Table S1, Additional file 1) were included in the control group (EH group). After excluding those with any prior diagnoses of any cause of dementia among the EH group, patients with PA (*n* = 3,687) were matched with EH controls (*n* = 14,741) based on age, sex, and index year (Fig. 1).

### Study outcomes

The primary outcomes were all-cause dementia events, including Alzheimer’s disease (AD, ICD-10 F00, or G30), vascular dementia (VD, ICD-10 F01), or other dementia (ICD-10 F02, or F03) combined with a prescription of one or more medications for dementia (donepezil, galantamine, memantine, or rivastigmine), which were well-validated in several studies using the NHIS cohort (see Supplementary Table S1, Additional file 1) [14, 15]. Each patient was followed up from the index date (date of the claim for ADX or MRA) to the earliest occurrence of any study outcomes, death, or the end of the observational period (December 31, 2017).

### Covariates

Income status was categorized into three groups (low 30%, middle 40%, and high 30%) based on the total national health insurance premiums paid by the insured individual in the index year. Patients were considered to have comorbidities (dyslipidemia, chronic kidney disease, AF, non-fatal stroke, and non-fatal myocardial infarction) if they had the presence of ICD-10 diagnostic codes (all available primary and secondary diagnoses) with relevant prescription medication use (prescribed > 30 accumulative days) before the index year (see Supplementary Table S1, Additional file 1). Concurrent medications such as antihypertensive agents, statins, and antithrombotic agents were also considered as confounders.

### Statistical analysis

Data are presented as means ± standard deviation (SD) for continuous variables and numbers (percentages) for categorical variables. Independent sample t-tests and chi-square tests analyzed the baseline characteristics of the patients. We used multivariable Cox regression models to evaluate the hazard ratios (HRs) and 95% confidence intervals (CIs) for the outcome incidence rates between patients with PA and their EH matches. Plots of Schoenfeld residuals confirmed no violation of the proportionality assumption over time. Due to the nature of the study duration, increased right censoring affected the survival curves, particularly after 10 years. This was due to fewer patients available for observation towards the end of the study. Models were adjusted for potential confounders. Model 1 was adjusted for age, sex, and income; model 2 was further adjusted for baseline comorbidities; model 3 was adjusted for prescribed medications. Kaplan–Meier survival curves were plotted, and the groups were compared using the log-rank test. In addition, subgroup analyses were conducted for age (≥ 65 and < 65 years), sex (male and female), the presence of pre-existing cardiovascular diseases (AF, non-fatal stroke, and non-fatal myocardial infarction), and DM to explore potentially susceptible groups for the onset of dementia in individuals with PA.

All analyses were conducted using SAS version 9.3 (SAS Institute, Cary, NC) and R version 4.0.1 (R Foundation for Statistical Computing, Vienna, Austria). Statistical significance was defined as a two-sided *p*-value < 0.05.

## Results

### Characteristics of study participants

PA (*n* = 3687) and EH (*n* = 14,741) groups did not differ in mean age (56 ± 10 years) and proportion of men (47%), with balanced entry to the cohort for each index year (Table 1). Compared to the EH group, individuals with PA had a higher prevalence of comorbidities, including DM (69% vs 40%), dyslipidemia (84% vs 61%), AF (11% vs 2%), and non-fatal stroke (9% vs 3%) or myocardial infarction (14% vs 4%) at baseline. As expected, the proportion of anti-hypertensive agent use was also higher in individuals with PA compared to the EH group. Among those with PA, the MRA group was older (58.8 versus 51.9 years, *p* < 0.001), included a higher proportion of men (50.3% versus 42.9%, *p* < 0.001), and had a higher prevalence of DM, dyslipidemia, AF, non-fatal myocardial infarction, and stroke as compared with the ADX group.

**Table 1:** Baseline characteristics

	**PA**			**EH**^**a**^	***p*-value**†
	**Total** **(*N* = 3687)**	**ADX** **(*N* = 1339)**	**MRA** **(*N* = 2348)**	**control** **(*N* = 14741)**	
Age, mean (SD)	56.27 ± 10.86	51.90 ± 8.06	58.77 ± 11.44^‡^	56.26 ± 10.83	0.936
Sex (male)	1755 (47.6)	574 (42.87)	1181 (50.3)	7019 (47.62)	0.986
Index year, n (%)					0.999
2003	67 (2.85)	49 (3.66)	116 (3.15)	460 (3.12)	
2004	92 (3.92)	55 (4.11)	147 (3.99)	588 (3.99)	
2005	93 (3.96)	54 (4.03)	147 (3.99)	588 (3.99)	
2006	89 (3.79)	52 (3.88)	141 (3.82)	564 (3.83)	
2007	113 (4.81)	77 (5.75)	190 (5.15)	757 (5.14)	
2008	144 (6.13)	76 (5.68)	220 (5.97)	880 (5.97)	
2009	129 (5.49)	88 (6.57)	217 (5.89)	868 (5.89)	
2010	165 (7.03)	95 (7.09)	260 (7.05)	1040 (7.06)	
2011	207 (8.82)	101 (7.54)	308 (8.35)	1232 (8.36)	
2012	173 (7.37)	108 (8.07)	281 (7.62)	1124 (7.62)	
2013	195 (8.30)	116 (8.66)	311 (8.44)	1244 (8.44)	
2014	258 (10.99)	123 (9.19)	381 (10.33)	1524 (10.34)	
2015	214 (9.11)	132 (9.86)	346 (9.38)	1384 (9.39)	
2016	283 (12.05)	146 (10.90)	429 (11.64)	1716 (11.64)	
2017	126 (5.37)	67 (5.00)	193 (5.23)	772 (5.24)	
Social economic status					
Lower 30%	1678 (46.32)	626 (47.5)	1052 (45.64)^‡^	5557 (38.56)	< 0.001
Middle 40%	1110 (30.64)	424 (32.17)	686 (29.76)^‡^	4985 (34.59)	< 0.001
Highest 30%	835 (23.05)	268 (20.33)	567 (24.6)^‡^	3868 (26.84)	< 0.001
Comorbidity, n (%)					
Diabetes	2540 (68.89)	838 (62.58)	1702 (72.49)^‡^	5896 (40)	< 0.001
Dyslipidemia	3095 (83.94)	1098 (82)	1997 (85.05)^‡^	8949 (60.71)	< 0.001
Chronic kidney disease	280 (7.59)	86 (6.42)	194 (8.26)	164 (1.11)	< 0.001
Atrial fibrillation	413 (11.2)	77 (5.75)	336 (14.31)^‡^	324 (2.2)	< 0.001
Non-fatal MI	524 (14.21)	111 (8.29)	413 (17.59)^‡^	624 (4.23)	< 0.001
Non-fatal stroke	331 (8.98)	95 (7.09)	236 (10.05)^‡^	505 (3.43)	< 0.001
Medication, n (%)					
ARB/ACE inhibitor	3090 (83.81)	1159 (86.56)	1931 (82.24)^‡^	9242 (62.7)	< 0.001
β-blocker	2788 (75.62)	1024 (76.47)	1764 (75.13)^‡^	3292 (22.33)	< 0.001
CCB	3291 (89.26)	1295 (96.71)	1996 (85.01)	8554 (58.03)	< 0.001
Diuretics	3567 (96.75)	1251 (93.43)	2316 (98.64)^‡^	5122 (34.75)	< 0.001
Statins	1687 (45.76)	512 (38.24)	1175 (50.04)^‡^	4898 (33.23)	< 0.001
Antithrombotics	1971 (53.46)	635 (47.42)	1336 (56.9)^‡^	3566 (24.19)	< 0.001

Values are presented as frequencies in numbers (percentages) or means (standard deviation)

*Abbreviations*: *ACE* angiotensin-converting enzyme, *ADX* adrenalectomy group, *ARB* angiotensin II receptor antagonists, *CCB* calcium channel blocker, *EH* essential hypertension, *MI* myocardial infarction, *MRA* mineralocorticoid receptor antagonist group, *PA* primary aldosteronism

^†^*p*-value for total PA group vs matched EH group

^‡^*p*-value < 0.05 vs. ADX group

^a^Cases and controls were matched by age, sex, and the index year

### Risk of dementia in primary aldosteronism from treatment initiation

**Fig. 2:**
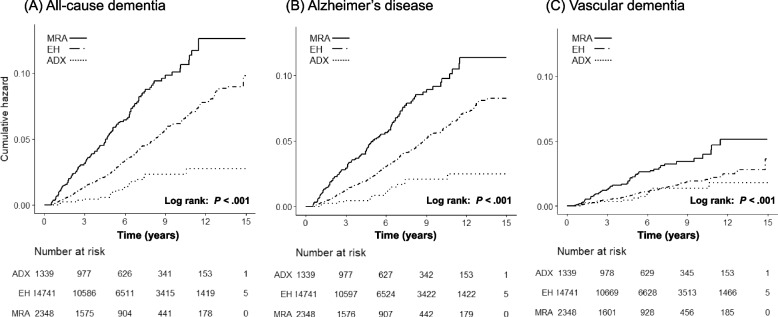
Kaplan–Meier survival curves for (**A**) all-cause dementia, (**B**) Alzheimer's disease, and (**C**) vascular dementia according to PA treatment groups (ADX group vs MRA group) compared to their EH matches after treatment initiation. Abbreviations: ADX indicates adrenalectomy; EH, essential hypertension; MRA, mineralocorticoid receptor antagonist; PA, primary aldosteronism

During a follow-up period that ranged from a minimum of 6 months to a maximum of 15 years, with a median follow-up of 5.2 years (interquartile range 2.7–8.7), all-cause dementia was newly diagnosed in 156 individuals of the PA group (Total: 156/3687, 4.2%; ADX: 18/1339, 1.3%; MRA: 138/2348, 5.9%) and 522 individuals of the EH group (522/14741, 3.5%). As shown in Table 2 and Fig. 2A, the incidence rate of all-cause dementia was higher in PA compared to the EH group (7.45 vs 5.92/1000 person-years; unadjusted HR 1.26, 95% CI 1.05–1.51) from the time point of any treatment initiation for PA. Compared with EH, the MRA group (*n* = 2348) had a higher risk of all-cause dementia (10.92/1000 person-years, unadjusted HR 1.88, 95% CI 1.56 to 2.27), whereas the ADX group (*n* = 1339) showed 65% lowered risk of all-cause dementia (2.16/1000 person-years; unadjusted HR 0.35, 95% CI 0.22 to 0.57). The association of PA with an elevated risk of dementia remained robust after adjustment for age, sex, and income (model 1) and further adjustment for baseline comorbidities (model 2; adjusted HR [aHR] 0.28 for ADX group; aHR 1.30 for MRA group). Although the MRA group was still associated with a higher risk of all-cause dementia after further adjustment for baseline medication uses (model 3; aHR 1.31, 95% CI 1.03 to 1.67), the statistical significance of the association between ADX and dementia was lost in the full model (aHR 0.69, 95% CI 0.41 to 1.15). When Alzheimer's disease and VD were analyzed separately, the statistical significance for the association of PA with Alzheimer's disease (ADX or MRA groups) was attenuated in the full model (Table 2 and Fig. 2B). However, the elevated risk of VD was observed in patients with PA compared to those with EH independent of age, sex, income, baseline comorbidities, and medication use (model 3; aHR 1.59, 95% CI 1.07 to 2.36), mainly driven by elevated risk in MRA groups (MRA, aHR 1.62, 95% CI 1.08 to 2.45; ADX, aHR 1.45, 95% CI 0.75 to 2.81) (Table 2 and Fig. 2C).

**Table 2:** Comparing risks for dementia between patients with PA and their EH matches after treatment initiation

	Number of events	Person-years	Cumulative incidence/1,000 person-years	Univariable Cox regression		Multivariable Cox regression^a^					
						Model 1		Model 2		Model 3	
				HR (95%CI)	*p*-value	HR (95%CI)	*p*-value	HR (95%CI)	*p*-value	HR (95%CI)	*p*-value
**All-cause dementia**											
EH (reference)	522	88,156	5.92	1.00		1.00		1.00		1.00	
PA (Total)	156	20,953	7.45	**1.26 (1.05–1.51)**	0.011	**1.26 (1.06–1.52)**	0.011	0.90 (0.74–1.10)	0.317	1.20 (0.95–1.51)	0.134
PA (ADX)	18	8317	2.16	**0.35 (0.22–0.57)**	< 0.001	**0.35 (0.22–0.57)**	< .0001	**0.28 (0.17–0.46)**	< 0.001	0.69 (0.41–1.15)	0.157
PA (MRA)	138	12,637	10.92	**1.88 (1.56–2.27)**	< 0.001	**1.87 (1.55–2.26)**	< .0001	**1.30 (1.06–1.61)**	0.013	**1.31 (1.03–1.67)**	0.027
**Alzheimer disease**											
EH (reference)	475	88,257	5.38	1.00		1.00		1.00		1.00	
PA (Total)	140	20,979	6.67	**1.24 (1.03–1.50)**	0.027	**1.24 (1.02–1.50)**	0.029	0.87 (0.71–1.08)	0.209	1.17 (0.91–1.49)	0.220
PA (ADX)	16	8322	1.92	**0.34 (0.20–0.56)**	< 0.001	**0.34 (0.20–0.56)**	< .0001	**0.27 (0.16–0.45)**	< 0.001	0.68 (0.40–1.18)	0.172
PA (MRA)	124	12,657	9.80	**1.85 (1.51–2.26)**	< 0.001	**1.84 (1.51–2.25)**	< .0001	**1.26 (1.01–1.58)**	0.039	1.27 (0.99–1.63)	0.062
**Vascular dementia**											
EH (reference)	169	89,192	1.89	1.00		1.00		1.00		1.00	
PA (Total)	65	21,207	3.06	**1.64 (1.23–2.18)**	0.001	**1.64 (1.23–2.19)**	0.001	1.16 (0.85–1.60)	0.352	**1.59 (1.07–2.36)**	0.020
PA (ADX)	12	8338	1.44	0.76 (0.42–1.37)	0.358	0.78 (0.43–1.40)	0.404	0.64 (0.35–1.16)	0.141	1.45 (0.75–2.81)	0.265
PA (MRA)	53	12,869	4.12	**2.21 (1.62–3.02)**	< 0.001	**2.19 (1.61–2.99)**	< 0.001	**1.48 (1.05–2.10)**	0.026	**1.62 (1.08–2.45)**	0.020

*Abbreviations*: *ADX* adrenalectomy, *CI* confidence interval, *EH* essential hypertension, *HR* hazard ratio, *MRA* mineralocorticoid receptor antagonist, *PA* primary aldosteronism

^a^Model 1: age, sex, and income; Model 2: model 1 + baseline comorbidities (diabetes mellitus, dyslipidemia, chronic kidney disease, atrial fibrillation, non-fatal stroke, and non-fatal myocardial infarction); Model 3: model 2 + prescribed medications (angiotensin II receptor antagonists/angiotensin-converting-enzyme inhibitor (ARB/ACE inhibitor), β-blocker, calcium channel blocker (CCB), diuretics, statins, and antithrombotics)

### Risk of dementia in primary aldosteronism from hypertension diagnosis

When the dementia risk was analyzed from the time point of initial hypertension diagnosis instead of treatment initiation, the association of the MRA group with a higher risk of all-cause dementia (aHR 1.54 in full model, *p* < 0.001) and VD (aHR 2.18, *p* < 0.001) remained robust (see Supplementary Table S2, Additional file 2). However, compared to individuals with EH, those with PA were also associated with a higher risk of all-cause dementia (aHR 1.45, *p* < 0.001) and Alzheimer's disease (aHR 1.47, *p* < 0.001). This was primarily attributed to the elevated risk in the MRA group (for all-cause dementia, aHR 1.54, *p* < 0.001; for Alzheimer's disease, aHR 1.55, *p* < 0.001), but the risk of dementia in the ADX group did not significantly differ from that of EH.

### Subgroup analysis

**Fig. 3:**
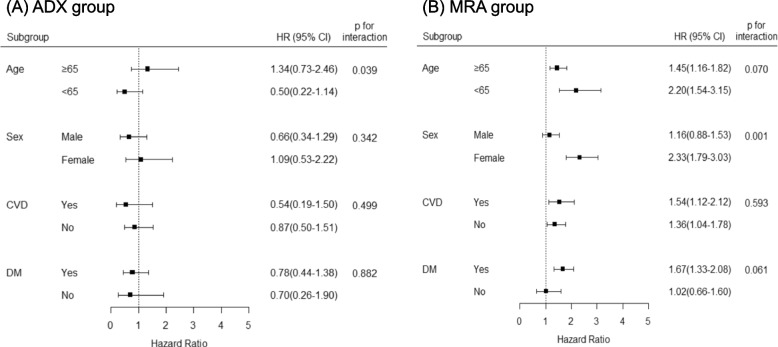
Subgroup analysis for the risk of all-cause dementia in PA treatment groups (**A**. ADX group, **B**. MRA group) compared to their EH matches. CVD includes myocardial infarction, stroke, and atrial fibrillation. Results have been adjusted for age, sex, and socioeconomic status. P for interaction is a statistical significance of effect modification by factors determining the subgroup. Abbreviations: ADX indicates adrenalectomy; CI, confidence interval; CVD, cardiovascular disease; DM, diabetes mellitus; EH, essential hypertension; HR, hazard ratio; MRA, mineralocorticoid receptor antagonist; PA, primary aldosteronism

Figure 3 shows the results of subgroup analyses for the association between PA treatment strategy (ADX and MRA) and dementia events in patients with PA. The risk of all-cause dementia did not differ between ADX and EH groups in all subgroups, with a beneficial effect of ADX in patients aged younger than 65 years compared to those aged 65 years or older (aHR 0.50, 95% CI 0.22 to 1.14 vs aHR 1.34, 95% CI 0.73 to 2.46, p for interaction = 0.039). For PA patients treated with MRA, a stronger association was observed for the increased risk of all-cause dementia in women (aHR 2.33 vs EH, 95% CI 1.79 to 3.03) than in men (aHR 1.16 vs EH, 0.88 to 1.53, p for interaction = 0.001). Compared to the EH group, the MRA group was associated with a higher risk of all-cause dementia in both older (aHR 1.45, 95% CI 1.16 to 1.82) and younger age groups (aHR 2.20, 95% CI 1.54 to 3.15; p for interaction = 0.070). Moreover, the elevated risk of dementia in the MRA group remained significant, particularly in individuals with DM (aHR 1.67, 95% CI 1.33 to 2.08) compared to those without DM (aHR 1.02, 95% CI 0.66 to 1.60). However, the interaction did not reach statistical significance (*p* = 0.061).

## Discussion

Over a median follow-up of 5.2 years, our study suggested a potential association between PA and an increased risk of dementia compared to EH, although this was not significant in all adjusted models. Among patients with PA, the ADX group had a lower or similar risk of all-cause dementia compared to EH controls, whereas the MRA group had a significantly elevated risk of all-cause dementia, particularly VD, even after adjusting for age, sex, socioeconomic status, baseline comorbidities, and medication use. The association remained robust when the risk of dementia was analyzed from the time of PA treatment initiation or from hypertension onset. Furthermore, among the MRA group, women were more susceptible to the increased risk of dementia than men, with the presence of DM and age under 65 as additional potential risk factors.

PA shows a higher cardiovascular risk than EH, independent of blood pressure level [3]. This is because direct genomic or non-genomic effects of aldosterone excess contribute to the acceleration of target organ damages, including left ventricular hypertrophy, myocardial fibrosis, endothelial dysfunction, and impaired kidney function [16, 17]. Experiments using rodent models have shown that MR agonist administration to the cerebral ventricle induced vasopressor effect and changes in behavioral or autonomic function, including mood or appetite, and stimulated sodium appetite [18, 19]. Aldosterone crosses the blood–brain barrier (BBB) in a detectable amount as a lipophilic hormone [20]. Although most MR-expressing brain cells are primarily occupied by glucocorticoids, not just aldosterone, at normal homeostasis, various sites throughout the brain, such as the hippocampus, express MR, including aldosterone-sensitive MR in the nucleus of the solitary tract [21]. Consequently, aldosterone excess may alter the homeostasis of aldosterone action in the brain and directly contribute to the increased risk of dementia observed here, independent of the systemic effect of aldosterone on blood pressure; this needs to be further studied.

The RAAS has been implicated in cognitive decline as evidenced by animal studies [11]. Notably, model mice with RAAS activation displayed reduced cerebral blood flow and heightened oxidative stress compared to control groups [22]. Importantly, RAAS not only exhibits systemic activation, leading to effects such as angiotensin II-mediated vasoconstriction, but also local activation within organs, including the brain [23, 24]. The localized RAAS is particularly noteworthy as it can precipitate end-organ damage independently of the systemic pathway. Recent human studies have also provided support for the notion that elevated levels of blood angiotensin-converting enzyme-1 (ACE-1) or angiotensin II are associated with cognitive function. This suggests that the renin-angiotensin system may play a role in the pathophysiological processes underlying dementia. [25, 26]. Consequently, it is critical to inhibit both local and systemic RAAS to prevent cognitive impairment [27]. Moreover, some RAAS inhibitors, including MRAs, have been observed to yield clinical benefits in preventing cognitive impairment. This effect is potentially facilitated by their ability to traverse a compromised blood–brain barrier, independent of their antihypertensive properties [11]. Iwanami et al. demonstrated that blocking MRs with eplerenone mitigated ischemic brain damage by augmenting cerebral blood flow and reducing oxidative stress [28]. Furthermore, MR blockers were found to diminish the size of cerebral infarcts in stroke-prone hypertensive rats, attributed to the down-regulation of epidermal growth factor receptor mRNA expression [29]. These findings support the role of MR blockers in preserving cognitive function, which is of critical importance for patients with aldosteronism who are at an elevated risk of dementia. In our study, the data indicating a higher risk of all-cause dementia in the MRA group should be interpreted cautiously. Given the evidence mentioned earlier, it is unlikely that MRA treatment itself is the direct cause of an increased risk of dementia.

Among individuals with PA, the MRA-treated group had a higher risk of dementia than EH, while the risk of dementia was not statistically different between the ADX-treated group and EH. Hypertension increases the risk of vascular cognitive impairment and Alzheimer's disease by compromising structural and functional integrity of the cerebral microcirculation, cerebromicrovascular endothelial dysfunction, and neurovascular uncoupling, leading to impaired cerebral blood supply [30]. Randomized clinical trials and individual-level meta-analysis of large cohorts indicated the beneficial effect of blood pressure lowering in mid-life on cognitive function [31]. MRA therapy was associated with less favorable long-term outcomes in patients with PA than ADX treatment, including a higher risk of mortality, AF, chronic kidney disease onset or progression, higher blood pressure after treatment initiation, and less improvement of quality of life [32–35]. Moreover, a longitudinal cohort study showed that individuals with PA treated with MRA who had unsuppressed renin (≥ 1 μg/L per h) had no significant excess risk compared to EH. In contrast, despite MRA use, those with suppressed renin levels had a significantly elevated risk of cardiovascular outcomes [32]. Likewise, suboptimal MR receptor blockade by MRA or non-compliance to medication in the MRA group could partly contribute to an elevated risk of dementia compared to EH or ADX groups. A study quantified white matter lesions (using T2-weighted magnetic resonance imaging [MRI], a precursor imaging marker of cerebral small vessel disease), BBB breakdown, and neuroinflammation leading to impaired brain function- using T2-weighted MRI. It reported that a lower plasma renin activity and higher plasma aldosterone concentration in patients with hypertension were associated with a higher white matter lesion load [10]. Therefore, further research is needed on whether an unsuppressed renin level in patients with PA on MRA therapy can serve as a clinical indicator of protection against the elevated risk of developing dementia.

A longer duration of hypertension is a major factor concerning an increased risk of worse outcomes suggesting cumulative neurological effects of high blood pressure [36]. According to the Primary Aldosteronism Surgical Outcome (PASO) investigators, patients diagnosed with hypertension for over 10 years tend to have less or little therapeutic effect from adrenalectomy compared to those with hypertension of 5 years among PA patients [37]. Furthermore, patients with PA with hypertension for a median of 8.8 years from the diagnosis of hypertension reportedly had increased risks of cardiovascular events and target organ damage compared to those with EH [3]. Similarly, our previous study also demonstrated a much higher risk of cardiovascular events from the time of hypertension diagnosis than treatment initiation for PA, depending on the longer duration of hypertension [13]. These results, together with our extended findings considering higher dementia risk among PA patients from the time of hypertension diagnosis, substantially highlight the clinical importance of early diagnosis and initiating proper treatment for PA for better outcomes.

Interestingly, we also found that the increased hazard of all-cause dementia in the MRA group was more prominent in women than in men. The PASO study showed that women with unilateral PA derived more clinical benefit from ADX, presumably related to the vasoprotective role of estrogen [37]. However, the underlying mechanism of the gender-specific differences in post-treatment outcomes of PA patients remains unclear. According to the Atherosclerosis Risk in Communities Study, middle-aged patients with hypertension had lower cognitive function scores than those without hypertension in women, not in men [38]. The relationship between midlife hypertension (mean age 44.3 years) and dementia risk was reportedly observed only among women, but not men [39]. Sex-specific effect of hypertension or arterial stiffness on white matter hyperintensities (WMHs) may provide another possible explanation. WMHs are considered among the main pathologies in VD and contribute to cognitive dysfunction in AD [40]. The association between hypertension and WMHs is reportedly stronger in women than in men [41]. Furthermore, pulse pressure, a marker of arterial stiffness, contributes to white matter structural damage more in women than in men [42]. Our results also suggest that relatively uncontrolled hypertension in the MRA group differentially affects the risk of dementia according to biological sex.

Furthermore, our findings highlight an age-dependent impact of treatment types on dementia risk in PA. Younger patients undergoing ADX appeared to exhibit a lower risk of dementia, which may be attributed to the removal of the source of excess aldosterone, thereby potentially mitigating aldosterone-induced vascular damage [37]. However, younger patients in the MRA group showed a higher, albeit statistically insignificant, risk of dementia. This aligns with studies indicating a correlation between high blood pressure and cognitive impairment in middle-aged patients [43]. Additionally, the co-existence of hypertension and DM further increases the risk of dementia. Chronic hyperglycemia is a well-known risk factor of cognitive dysfunction, explained by oxidative stress, accumulation of advanced glycation end products, extensive leukoaraiosis, and atrophy in the region of the hippocampus and amygdala [44]. Among PA treatment strategies, spironolactone treatment was associated with poor glucose metabolism compared to adrenalectomy. This is based on a positive correlation between insulin resistance and post-treatment plasma aldosterone concentration [45]. These findings reflect the necessity of optimal control of blood pressure and glucose level in younger and diabetic PA patients to prevent the development of dementia.

We believe this is the first study investigating the link between PA and incident dementia risk. The major strength of this study was the large national sample size, which provided sufficient statistical power and minimized selection. Furthermore, our analysis of dementia risk regarding the PA treatment strategy can enrich the scope of research in this field. Additionally, a detailed analysis considering two index points from the time of hypertension diagnosis and after medical or surgical treatment initiation revealed consistent results supporting the association between PA patients and dementia risks.

### Limitations

However, there are several limitations. First, a causal relationship between PA and incident dementia cannot be established as with all retrospective studies. Although we adjusted various covariates, residual confounding due to the observational study design may exist. Importantly, certain potentially relevant covariates such as years of education and lifestyle factors such as e smoking habits, regular exercise, and crucially, genetic factors like APOE-ε4, which are associated with dementia risk, were not available in the Korean nationwide cohort data we used. This lack of data might lead to residual confounding, even though we adjusted for numerous other relevant covariates. Second, the follow-up duration was relatively short to evaluate dementia outcomes because the mean age of our patients was younger than in other dementia studies. Third, we defined dementia outcomes based on the Korean NHIS claim datasets rather than the results of cognitive assessment tools, so we cannot rule out any inaccurate diagnosis. With regard to its subtyping, the differentiation between dementia subtypes such as AD, VD, and Lewy Body Dementia is inherently challenging due to the limitations of clinical assessments in the nationwide cohort data. Furthermore, the presence of mixed dementia with overlapping pathologies complicates accurate categorization, and definitive diagnoses through autopsy are not feasible in this study [14]. Furthermore, due to the nature of nationwide claim data, there may be limitations in the accuracy of the diagnosis for the Alzheimer’s disease, which can be influenced by factors such as healthcare policies and insurance coverage for anti-dementia medications. This necessitates cautious interpretation of the findings in the context of real-world clinical practice. Nevertheless, we considered the operational definition for dementia with diagnostic and prescription codes, which has been well validated in previous studies [14, 15]. Additionally, the accuracy of the PA diagnosis may not be secure since we used operational definitions based on claim databases. This cohort did not have data serum aldosterone level and plasma renin activity in evaluating the state of hyperaldosteronism. An inherent limitation of this study is the lack of information on the levels of blood pressure in the participants. This also limits an examination of potential differences in the degree of blood pressure elevation between the PA and EH groups, which could have implications for the interpretation of the association between these conditions and dementia risk. Future studies with detailed blood pressure data would be valuable to elucidate this associations observed in our study. Lastly, we acknowledge the inherent challenge in distinguishing individuals with prodromal dementia at baseline, a factor that could potentially impact our findings. A sensitivity analysis to exclude these prodromal stages could have provided more precise results; however, strict NHIS-HEALS regulations precluded this level of data exploration and additional statistical validation.

## Conclusions

The current study provides potential evidence that the risk of dementia may be higher in patients with PA, especially in the MRA-treated group, compared to those with EH. Females, younger age, and DM could be additional risk factors for incident dementia in the MRA group. The risk of all-cause dementia remained elevated compared to EH even after initiation of PA treatment, particularly in the MRA group. Therefore, long-term prospective studies to investigate the link between PA and dementia, including the effects of PA treatment strategies on incident dementia, are still warranted.

### Supplementary Information

**Additional file 1:** **Supplementary Table S1.** Definitions and codes used for defining key conditions, comorbidities, and drug treatments in this study.

**Additional file 2:** **Supplementary Table S2. **Comparison of risks for dementia outcomesbetween patients with PA and their EH matches from the time of initialhypertension diagnosis.

## Data Availability

The data underlying this article were provided by the Korean National Health Insurance Service by permission. Data will be shared on request to the corresponding author with permission of the Korean National Health Insurance Service.

## Abbreviations

AD: Alzheimer’s disease
ADX: Adrenalectomy
AF: Atrial fibrillation
aHR: Adjusted hazard ratio
BBB: Blood-brain barrier
CI: Confidence interval
DM: Diabetes mellitus
EH: Essential hypertension
HR: Hazard ratio
ICD-10: International classification of disease, 10th revision
MRA: Mineralocorticoid receptor antagonist
MRI: Magnetic resonance imaging
NHIS: National health insurance service
PA: Primary aldosteronism
PASO: Primary aldosteronism surgical outcome
SD: Standard deviation
VD: Vascular dementia
WMHs: White matter hyperintensities
