# Supplementary material for: Risk of dementia in primary aldosteronism compared with essential hypertension: a nationwide cohort study
Source: Alzheimers Res Ther. 2023 Aug 11;15:136. doi: 10.1186/s13195-023-01274-x (PMC10416485; doi:10.1186/s13195-023-01274-x)
Supplement: Supplementary file 1 — Additional file 1: Supplementary Table S1. Definitions and codes used for defining key conditions, comorbidities, and drug treatments in this study. [file 13195_2023_1274_MOESM1_ESM.docx]

**Additional File 1**

File format: Docx

Title: **Supplementary Table S1**. Definitions and codes used for defining key conditions, comorbidities, and drug treatments in this study

**Supplementary Table S1.** Definitions and codes used for defining key conditions, comorbidities, and drug treatments in this study

|  | **Definitions** | **ICD-10 Codes or conditions** |
| --- | --- | --- |
| **Key conditions** | |  |
| Dementia | Defined from a diagnostic code of dementia plus antidementia drugs at least once | ICD-10: Alzheimer’s disease (F00 or G30), Vascular dementia (F01), other dementia (F02 or F03)  Treatment: all kinds of  antidementia drugs (donepezil, rivastigmine, galantamine, memantine) |
| Essential hypertension | Defined from diagnosis at least twice a year plus treatment (≥180 days) | ICD-10: I10  Treatment: all kinds of antihypertensive agents (ARB, ACE inhibitors, beta-blockers, calcium channel blockers, alpha-blockers, diuretics, others) |
| **Comorbidities** | |  |
| Diabetes mellitus | Defined from diagnosis plus treatment (≥ 30 days) | ICD-10: E10-E14  Treatment: all kinds of oral antidiabetics and insulin. |
| Dyslipidemia | Defined from diagnosis plus treatment (≥ 30 days) | ICD-10: 78  Treatment: all kinds of lipid-lowering agents |
| Chronic kidney disease | Defined from diagnosis or any procedure code for kidney replacement treatment | ICD-10: N18, N18.5, Z94.0  Claims for kidney replacement treatment (Z49.1-49.2, O7072, O7073-7034, O7071, O7072, R3280) |
| Atrial fibrillation | Defined from diagnosis* | ICD-10: I48 |
| Non-fatal stroke | Defined from a diagnostic code of stroke plus a procedure code of brain image | ICD-10: I60-I66, I69.0-I69.2  Claims for brain image (HE101, HE102, HE135, HE136, HE201, HE202, HE235, HE236, HE301, HE302, HE501, HE502, HE535, HE536) |
| Non-fatal MI | Defined from a diagnostic code of MI plus a procedure code of coronary artery angiography | ICD-10: I20-I25,  Claim for Coronary revascularization (HA607, E0721, E0723, O1640, O1641, O1642, O1647, O1648, O1649, OA640, OA641, OA642, OA647, OA648, OA649, M6551, M6552, M6553, M6554, M6561, M6562, M6563, M6564, M6565, M6566, M6567, M6571, M6572) |
| **Medication (available in South Korea)** | | |
| ARB |  | azilsartan, candesartan, eprosartan, fimasartan, irbesartan, losartan, olmesartan, telmisartan, valsartan |
| ACE inhibitor |  | alacepril, captopril, cilazapril, enalapril, imidapril, lisinopril, perindopril, ramipril, temocapril, zofenopril |
| Beta-blocker |  | atenolol, carvedilol, metoprolol, nebivolol, S-atenolol, S-carvedilol |
| Calcium channel blocker |  | amlodipine, S-amlodipine, barnidipine, benidipine, cilnidipine, diltiazem, felodipine, flunarizine isradipine, lacidipine, lercanidipine, manidipine, nicardipine, nifedipine, nimodipine, nisoldipine, verapamil |
| Alpha-blockers |  | Bevantolol, Doxazosin, Phenoxybenzamine |
| Diuretics |  | amiloride, chlorthalidone, furosemide, hydrochlorothiazide, indapamide, metolazone, torsemide, xipamide |
| Statins |  | atorvastatin, fluvastatin, lovastatin, pravastatin sodium, pitavastatin, rosuvastatin, simvastatin |
| Antithrombotic agents |  | anagrelide, aspirin, apixaban, cilostazol, clopidogrel, dabigatran, edoxaban, ozagrel, prasugrel, rivaroxaban, sarpogrelate, ticagrelor, ticlopidine, warfarin |

ACE inhibitor, angiotensin-converting-enzyme inhibitor; ARB, angiotensin II receptor antagonists; CVD, cardiovascular disease; ICD-10, International Classification of Diseases, 10th revision; MI, myocardial infarction

*To ensure accuracy, comorbidities were established based on one inpatient or two outpatient records of ICD-10 codes in the database
