# Supplementary material for: Risk of dementia in primary aldosteronism compared with essential hypertension: a nationwide cohort study
Source: Alzheimers Res Ther. 2023 Aug 11;15:136. doi: 10.1186/s13195-023-01274-x (PMC10416485; doi:10.1186/s13195-023-01274-x)
Supplement: Supplementary file 2 — Additional file 2: Supplementary Table S2. Comparison of risks for dementia outcomesbetween patients with PA and their EH matches from the time of initialhypertension diagnosis. [file 13195_2023_1274_MOESM2_ESM.docx]

**Supplementary Table S2.** Comparison of risks for dementia outcomes between patients with PA and their EH matches from the time of initial hypertension diagnosis.

|  | Number of events | Person-years | Cumulative incidence | Univariable Cox regression | | Multivariable Cox regression* | | | | | |
| --- | --- | --- | --- | --- | --- | --- | --- | --- | --- | --- | --- |
|  |  |  |  |  |  | Model 1 | | Model 2 | | Model 3 | |
|  |  |  |  | HR  (95%CI) | p-value | HR  (95%CI) | p-value | HR  (95%CI) | p-value | HR  (95%CI) | p-value |
| **All-cause dementia** | | | | | | | | | | | |
| EH (reference) | 522 | 88156 | 5.92 | 1.00 |  | 1.00 |  | 1.00 |  | 1.00 |  |
| PA (Total) | 329 | 42212 | 7.79 | 1.11(0.96-1.28) | 0.172 | 1.11(0.96-1.29) | 0.150 | 1.15(0.99-1.33) | 0.069 | **1.45(1.24-1.69)** | <0.001 |
| PA (ADX) | 29 | 14016 | 2.07 | **0.28(0.19-0.42)** | <0.001 | **0.29(0.19-0.42)** | <0.001 | **0.31(0.21-0.45)** | <0.001 | 0.84(0.57-1.26) | 0.405 |
| PA (MRA) | 300 | 28196 | 10.64 | **1.52(1.30-1.76)** | <0.001 | **1.53(1.31-1.77)** | <0.001 | **1.54(1.32-1.79)** | <0.001 | **1.54(1.31-1.8)** | <0.001 |
| **Alzheimer disease** | | | | | | | | | | | |
| EH (reference) | 475 | 88257 | 5.38 | 1.00 |  | 1.00 |  | 1.00 |  | 1.00 |  |
| PA (Total) | 301 | 42279 | 7.12 | 1.12(0.96-1.3) | 0.148 | 1.13(0.97-1.32) | 0.123 | 1.17(1.00-1.37) | 0.051 | **1.47(1.25-1.73)** | <0.001 |
| PA (ADX) | 27 | 14022 | 1.93 | **0.29(0.2-0.43)** | <0.001 | **0.29(0.20-0.44)** | <0.001 | **0.32(0.21-0.47)** | <0.001 | 0.89(0.59-1.35) | 0.584 |
| PA (MRA) | 274 | 28257 | 9.70 | **1.54(1.32-1.8)** | <0.001 | **1.55(1.32-1.81)** | <0.001 | **1.56(1.33-1.83)** | <0.001 | **1.55(1.32-1.83)** | <0.001 |
| **Vascular dementia** | | | | | | | | | | | |
| EH (reference) | 169 | 89192 | 1.89 | 1.00 |  | 1.00 |  | 1.00 |  | 1.00 |  |
| PA (Total) | 150 | 42939 | 3.49 | **1.63(1.29-2.06)** | <0.001 | **1.62(1.29-2.05)** | <0.001 | **1.65(1.30-2.10)** | <0.001 | **2.09(1.63-2.69)** | <0.001 |
| PA (ADX) | 18 | 14075 | 1.28 | **0.60(0.36-0.97)** | 0.039 | **0.61(0.37-0.99)** | 0.048 | 0.66(0.40-1.08) | 0.101 | 1.56(0.93-2.62) | 0.095 |
| PA (MRA) | 132 | 28864 | 4.57 | **2.12(1.66-2.69)** | <0.001 | **2.11(1.66-2.68)** | <0.001 | **2.08(1.63-2.66)** | <0.001 | **2.18(1.69-2.81)** | <0.001 |

Abbreviations: ADX, adrenalectomy; CI, confidence interval; EH, essential hypertension; HR, hazard ratio; MRA, mineralocorticoid receptor antagonist; PA, primary aldosteronism.

*Model 1: age, sex, and income; Model 2: model 1 + baseline comorbidities (diabetes mellitus, dyslipidemia, chronic kidney disease, atrial fibrillation, non-fatal stroke, and non-fatal myocardial infarction); Model 3: model 2 + prescribed medication (angiotensin II receptor antagonists/angiotensin-converting-enzyme inhibitor (ARB/ACE inhibitor), β-blocker, calcium channel blocker (CCB), diuretics, statins, and antithrombotics)
